# Competing endogenous RNA networks related to prognosis in chronic lymphocytic leukemia: comprehensive analyses and construction of a novel risk score model

**DOI:** 10.1186/s40364-022-00423-y

**Published:** 2022-10-21

**Authors:** Xin Zhang, Yang Han, Xinting Hu, Hua Wang, Zheng Tian, Ya Zhang, Xin Wang

**Affiliations:** 1 Department of Hematology, Shandong Provincial Hospital, Shandong University, No.324, Jingwu Road, Jinan, 250021 Shandong China; 2 Department of Hematology, Shandong Provincial Hospital Affiliated to Shandong First Medical University, No.324, Jingwu Road, Jinan, 250021 Shandong China; 3 Shandong Provincial Engineering Research Center of Lymphoma, Jinan, 250021 Shandong China; 4 Branch of National Clinical Research Center for Hematologic Diseases, Jinan, 250021 Shandong China; 5 National Clinical Research Center for Hematologic Diseases, the First Affiliated Hospital of Soochow University, Suzhou, 251006 China

**Keywords:** Chronic lymphocytic leukemia, Competing endogenous RNA, Risk score model, Prognostic biomarkers, Non-coding RNAs

## Abstract

**Background:**

Chronic lymphocytic leukemia (CLL) is a heterogeneous B-cell malignancy that lacks specific biomarkers and drug targets. Competing endogenous RNAs (ceRNAs) play vital roles in oncogenesis and tumor progression by sponging microRNAs (miRNAs). Nevertheless, the regulatory mechanisms of survival-related ceRNA networks in CLL remain to be uncovered.

**Methods:**

We included 865 de novo CLL patients to investigate RNA expression profiles and Illumina sequencing was performed on four CLL patients, two CLL cell lines and six healthy donors in our center. According to univariate Cox regression, LASSO regression as well as multivariate Cox regression analyses, we established a novel risk score model in CLL patients. Immune signatures were compared between the low- and high-risk groups with CIBERSORT and ESTIMATE program. Afterwards, we analyzed the relationship between differentially expressed miRNAs (DEmiRNAs) and IGHV mutational status, p53 mutation status and del17p. Based on the survival analyses and differentially expressed RNAs with targeting relationships, the lncRNA/circRNA-miRNA-mRNA ceRNA networks were constructed. In addition, the circRNA circ_0002078/miR-185-3p/TCF7L1 axis was verified and their interrelations were delineated by dual-luciferase reporter gene assay.

**Results:**

Totally, 57 differentially expressed mRNAs (DEmRNAs) and 335 DEmiRNAs were identified between CLL patient specimens and normal B cells. A novel risk score model consisting of HTN3, IL3RA and NCK1 was established and validated. The concordance indexes of the model were 0.825, 0.719 and 0.773 in the training, test and total sets, respectively. The high-risk group was related to del(13q14) as well as shorter overall survival (OS). Moreover, we identified DEmiRNAs that related to cytogenetic abnormality of CLL patients, which revealed that miR-324-3p was associated with IGHV mutation, p53 mutation and del17p. The survival-related lncRNA/circRNA-miRNA-mRNA ceRNA networks were constructed to further facilitate the development of potential predictive biomarkers. Besides, the expression of circ_0002078 and TCF7L1 were significantly elevated and miR-185-3p was obviously decreased in CLL patients. Circ_0002078 regulated TCF7L1 expression by competing with TCF7L1 for miR-185-3p.

**Conclusions:**

The comprehensive analyses of RNA expression profiles provide pioneering insights into the molecular mechanisms of CLL. The novel risk score model and survival-related ceRNA networks promote the development of prognostic biomarkers and potential therapeutic vulnerabilities for CLL.

**Supplementary Information:**

The online version contains supplementary material available at 10.1186/s40364-022-00423-y.

## Background

As the most prevalent type of leukemia among adults in the western world [1], chronic lymphocytic leukemia (CLL) is defined as a monoclonal lymphoproliferative disease featuring the gradual cumulation of immunologically dysfunctional mature B lymphocytes [2, 3]. Even if there have been great advances in the diagnosis and treatment of CLL, the underlying pathogenesis of CLL has not been completely understood until now. With the development of RNA-sequencing, a myriad of potential biomarkers in association with the prognosis of CLL have been discovered. However, we still need to explore risk score models with highly robust predictive power for survival to provide a basis for individualized treatment of CLL patients.

Lately, accumulative evidence revealed that non-coding RNAs (ncRNAs) played pivotal roles in regulating gene expression [4, 5] and can affect the cancer process by regulating alternative splicing [6, 7]. NcRNAs, which are a group of RNAs that do not have protein coding function, contain many categories. Among them, microRNA (miRNA), long noncoding RNA (lncRNA) and circular RNA (circRNA) are mainly involved in post-transcriptional regulation [8].

Competing endogenous RNAs (ceRNAs) hypothesis was first proposed by the team of Pier Paolo Pandolfi, which assumed that if lncRNAs or other types of RNA molecules have the same miRNA response elements (MREs) as mRNA, they can act as ceRNAs to compete with mRNA for binding to miRNAs, accordingly influencing the regulation of miRNAs on its target genes [9]. This hypothesis has been widely accepted and emerging evidence has showed the roles of ceRNA networks in tumorigenesis, invasion and metastasis [10, 11]. For example, lncRNA TMPO-AS1/miR-126-5p/BRCC3 axis facilitates gastric carcinoma development and angiogenesis [12]. LncRNA APCDD1L-AS1 can suppress autophagic degradation of EGFR through miR-1322/miR-1972/miR-324-3p-SIRT5 axis, thereby contributes to icotinib resistance [13]. The inhibition of lncRNA LAMP5-AS1 dramatically decreases colony formation and enhances primary MLL leukemia CD34^+^ cells differentiation [14]. Wang et al. found that in hepatocellular carcinoma, circ_0001588/miR-874/CDK4 aixs promoted cell proliferation, invasion, and migration [15]. CircRNA circ_0025033/miR-184/LSM4 axis was shown to accelerate the development of ovarian cancer [16]. In colorectal cancer, circHERC4 inactivates miR-556-5p, resulting in the activation of the CTBP2/E-cadherin pathway, which promotes cancer metastasis [17].

In CLL, the functions of certain lncRNA/circRNA axes in the development of CLL have been elucidated. Circ-CBFB was highly expressed in CLL and promoted FZD3 expression by inhibiting miR-607, resulting in the activation of the Wnt/β-catenin pathway and subsequent CLL progression [18]. Circ_0132266 was found to accelerate apoptosis and inhibit proliferation by miR-337-3p/PML pathway [19]. In addition, Li et al. found that the relative expression of circ-RPL15 in CLL patients was up-regulated and was positively associated with IGHV mutation status. Circ-RPL15 can sponge miR-146b-3p and block its suppression of the RAS/RAF1/MEK/ERK pathway [20]. LncRNA CRNDE was down-regulated in CLL cell lines, and its protective effect in CLL was to impair proliferation through the miR-28/NDRG2 axis [21]. Yet, the roles of the ceRNA networks in CLL and their regulatory mechanism as well as more meaningful biomarkers still require further exploration.

In the present study, distinct RNA expression signatures were identified in CLL patients and CLL cell lines. Differentially expressed genes (DEGs) in association with the overall survival (OS) were revealed, and a novel risk score model including HTN3, IL3RA and NCK1 was established. Besides, we constructed survival-related ceRNA networks and validated the circRNA circ_0002078/miR-185-3p/TCF7L1 axis. Our results provide an in-depth understanding of the underlying mechanism of CLL pathogenesis. Moreover, the current study is helpful for uncovering novel prognostic biomarkers and therapeutic vulnerabilities of CLL.

## Materials and methods

### Patient specimens

In this study, 865 de novo CLL patients were included. The CLL patients for Illumina sequencing were collected from Shandong Provincial Hospital CLL (SPHCLL) database and were diagnosed on the basis of the revised International Workshop on Chronic Lymphocytic Leukemia criteria [22]. B cells of these samples were separated according to the previously reported method [23]. Ethics approval was acquired from the Medical Ethical Committee of Shandong Provincial Hospital, and all of the patients and healthy donors have given their informed consent.

### RNA sequence analyses and differentially expressed analyses

The lncRNA, circRNA, miRNA and mRNA expression data of normal B cells, CLL patient specimens and CLL cells were available from Illumina sequencing. Compare CLL patient specimens and CLL cells with normal B cells, we identified the differentially expressed mRNAs (DEmRNAs), differentially expressed miRNAs (DEmiRNAs), differentially expressed lncRNAs (DElncRNAs) and differentially expressed circRNAs (DEcircRNAs) with the statistical threshold of |log_2_fold change|> 2 as well as *p* values < 0.05.

### Functional enrichment analyses

Gene Ontology (GO) and Kyoto Encyclopedia of Genes and Genomes (KEGG) analyses were performed to analyze the molecular function (MF), cell component (CC), biological processes (BP) and signaling pathways of DEmRNAs. *P* values < 0.05 were regarded as statistically significant.

### Construction of the novel risk score model

Univariate Cox regression analysis was applied to initially filter DEGs in association with OS. LASSO regression analysis was conducted on these DEGs to avoid overfitting. Multivariate Cox regression method was used to establish the risk score model. The calculation method of the risk scoring system is as follows: Risk score = coefficient_1_ * gene_1_ expression value + coefficient_2_ * gene_2_ expression value + coefficient_i_ * gene_i_ expression. The above analyses were conducted by R software.

### Exploration of immune signatures between low-risk and high-risk groups

In order to explore the distinct immune signatures between low-risk and high-risk groups, we performed the CIBERSORT algorithm. The ESTIMATE method was conducted to compute the scores of immune and stromal cells as well as ESTIMATE score in low-risk and high-risk groups.

### Construction of lncRNA/circRNA-miRNA-mRNA ceRNA networks

We used miRanda and psRobot software to constructed ceRNA regulatory networks with DElncRNA/DEcircRNA as decoy, DEmiRNA as the core, and DEmRNA as the target according to the RNA sequence results. Cytoscape 3.7.1 was used to visualize and select the lncRNA/circRNA-miRNA-mRNA ceRNA networks on the basis of the DEGs that associated with OS.

### Cell lines and cell culture

The cell lines for RNA-sequencing were MEC1 cell line and EHEB cell line. The MEC1 cells were obtained from University of California, San Diego. The EHEB cells and 293 T cells were purchased from American type culture collection. IMDM with 10% fetal bovine serum (FBS) was the medium for culturing the MEC1 cells. The EHEB cells and 293 T cells were cultured in RPMI1640 and DMEM respectively with FBS (10%). Cell culture conditions are 37 °C, 5% CO_2_ and saturated humidity.

### Quantitative real-time polymerase chain reaction (qRT-PCR)

After the ceRNA networks were established, we validated three DEGs (TRIM34, SLC30A10, HOXD4), circ_0007675 and the circ_0002078/miR-185-3p/TCF7L1 axis. Peripheral blood mononuclear cells of patients with CLL and healthy donors were extracted following the previous method [24, 25]. Total RNA was obtained by RNAiso Plus (TaKaRa, Dalian, China). Then reverse transcription reagents (Accurate Biology, Dalian, China) were used for reverse transcription reaction to obtain cDNA. The qRT-PCR was performed with SyberGreen (Accurate Biology, Changsha, China) in LightCycler 480II (Roche, Basel, Swizerland). Internal controls were U6 and GAPDH. Biosune (Shanghai, China) synthesized all of the primers. Relevant primer sequences are shown in Supplementary table S9.

### Dual-luciferase reporter gene assay

The interrelations between circ_0002078 and miR-185-3p, miR-185-3p and TCF7L1 were confirmed through the dual-luciferase reporter gene assay. PmirGLO-circ_0002078-WT/Mut and pmirGLO-3′UTR TCF7L1-WT/Mut plasmids (constructed by Biosune (Shanghai, China)) were co-transfected with miR-185-3p mimics and miR-NC respectively into the 293 T cells using lipofectamine 3000 (Invitrogen, USA). Dual-Luciferase Assay System Kit (E1910, Promega, USA) was applied to detect the relative luciferase activity after 48 h.

### Cell proliferation assays

Cell proliferation was assessed with cell Counting Kit-8 (CCK-8; YEASEN, Shanghai, China). A total of 1 × 10^4^ MEC1 or EHEB cells (each well) were seeded in a 96-well culture plate. After 24, 48, and 72 h of incubation, 10 μl of CCK-8 solution was added to each well. The absorbance of each well was measured at 450 nm.

### Analysis of cell apoptosis and cell cycle

PE Annexin V Apoptosis Detection Kit I (BD Pharmingen, Bedford, MA, USA) was used for the analysis of cell apoptosis. After collecting treated cells, resuspended the cells and added 5 μl each of Annexin V-PE and 7AAD and incubated the mixture at room temperature for 15 min. Cells for cell cycle analysis were soaked in 70% ethanol overnight at 4 °C and then incubated with PI/ RNase Staining Buffer (BD Biosciences, Bedford, MA, USA) for 15 min. Navios Flow Cytometer (Beckman Coulter, CA, USA) was used for cell apoptosis and cell cycle analyses.

### Statistical analyses

Kaplan–Meier (K-M) method was performed to generate survival curves. For the purpose of quantitatively evaluating the prognosis of CLL, we used the “rms” R package to establish the prognostic DEGs nomogram. The relevance between risk score model and cytogenetic abnormalities were explored by Chi-square test. The relationship between gene expression and clinical or other characteristics were identified by T-test. the relationship between circ_0002078 and TCF7L1 was explored by Pearson correlation analysis. *P* values < 0.05 were considered to be statistically significant.

## Results

### Differentially expressed RNAs between CLL patients or CLL cells and normal B cells

**Fig. 1:**
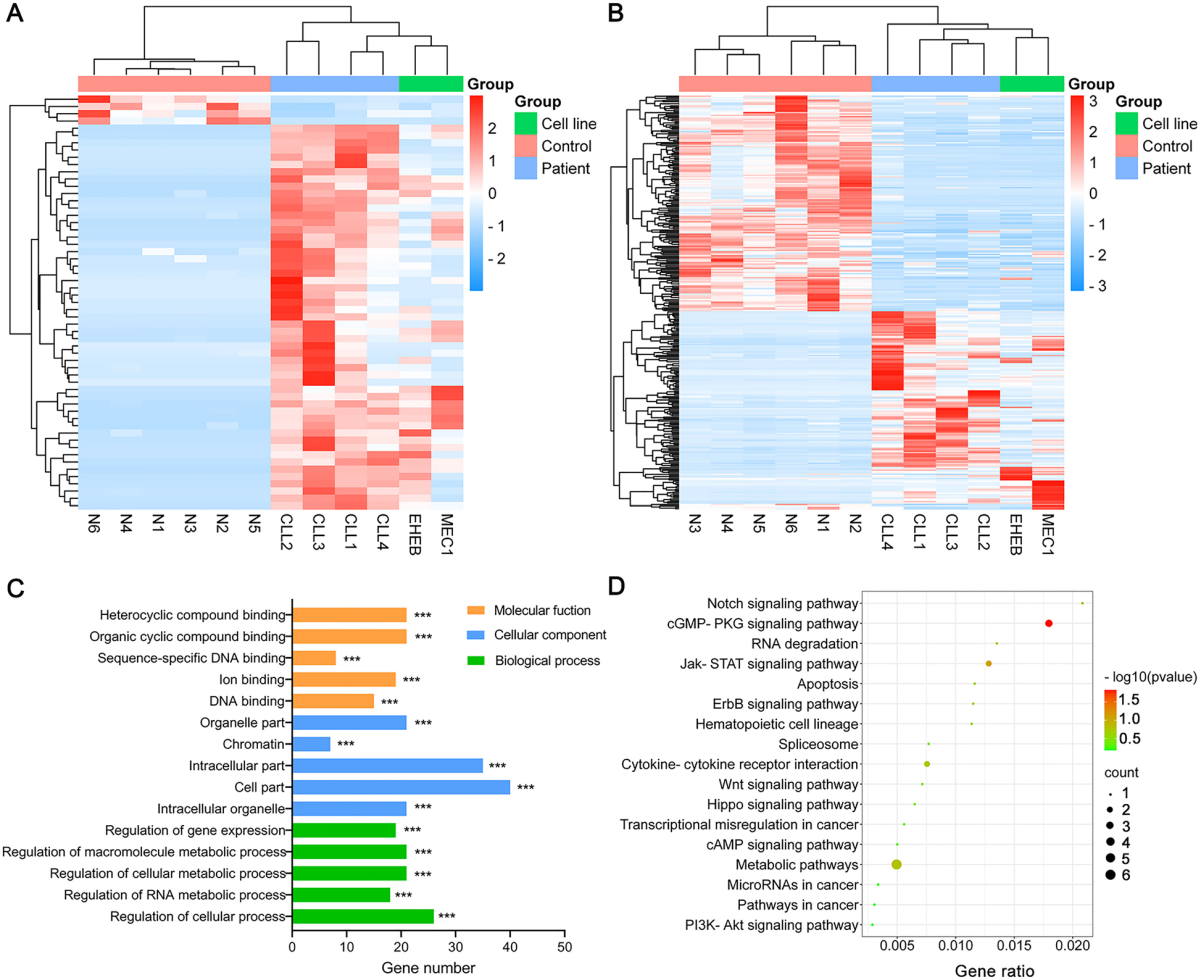
The heatmap and functional enrichment analyses in chronic lymphocytic leukemia (CLL) patients. **A** The heatmap of differentially expressed miRNAs in CLL patients, CLL cell lines and control. **B** The heatmap of differentially expressed mRNAs in CLL patients, CLL cell lines and control. **C** GO analysis results showed that changes in molecular function (MF), cell component (CC) and biological processes (BP) of DEGs between CLL patients and control were mainly enriched in heterocyclic compound binding, organelle part and regulation of gene expression. **D** KEGG pathway analysis revealed that the DEGs between CLL patients and control were mainly enriched in Notch signaling pathway and JAK-STAT signaling pathway

The DElncRNAs, DEcircRNAs, DEmiRNAs and DEmRNAs between CLL patient specimens, CLL cells and normal B cells were acquired from the Illumina sequencing. Totally, we identified 57 DEmRNAs and 335 DEmiRNAs by comparing CLL patient specimens with normal B cells. At the same time, between CLL cells and normal B cells, 482 mRNAs and 302 miRNAs were showed differential expression. DEmRNAs and DEmiRNAs in the samples were shown in the heatmap (Fig. 1A-B). The top 10 up-regulation and down-regulation differentially expressed RNAs were presented in Supplementary table S1-8.

### Functional enrichment analyses of DEGs

The potential functions of DEGs were explored by GO and KEGG analyses. GO analysis results demonstrated that changes in MF of DEGs between CLL patients and healthy controls were significantly focused on heterocyclic compound binding, organic cyclic compound binding and sequence-specific DNA binding (Fig. 1C). Meanwhile, the changes in MF of DEGs between MEC1 and EHEB cell lines and controls were significantly focused on sequence-specific DNA binding, oxidoreductase activity and cofactor binding (Supplementary Fig. 1A). Changes in CC of DEGs between CLL patient specimens and healthy donors were principally involved in organelle part, chromatin and intracellular part (Fig. 1C), meanwhile, between CLL cells and healthy controls they were involved in Golgi membrane, ribosomal subunit and Golgi apparatus part (Supplementary Fig. 1A). Changes in BP of DEGs between CLL patients and healthy donors primarily included regulation of gene expression, regulation of macromolecule metabolic process and regulation of cellular metabolic process (Fig. 1C), which between CLL cells and healthy controls primarily included cation transmembrane transport, lipid metabolic process and negative regulation of RNA metabolic process (Supplementary Fig. 1A).

For KEGG pathway analysis, the DEGs between CLL specimens and healthy donors were primarily relevant to Notch signaling pathway, JAK-STAT signaling pathway and cGMP-PKG signaling pathway (Fig. 1D). Additionally, between CLL cell lines and normal groups, DEGs were significantly associated with mTOR signaling pathway, NF-kappaB signaling pathway and cell cycle  (Supplementary Fig. 1B).

### Construction and validation of a novel risk score model

**Fig. 2:**
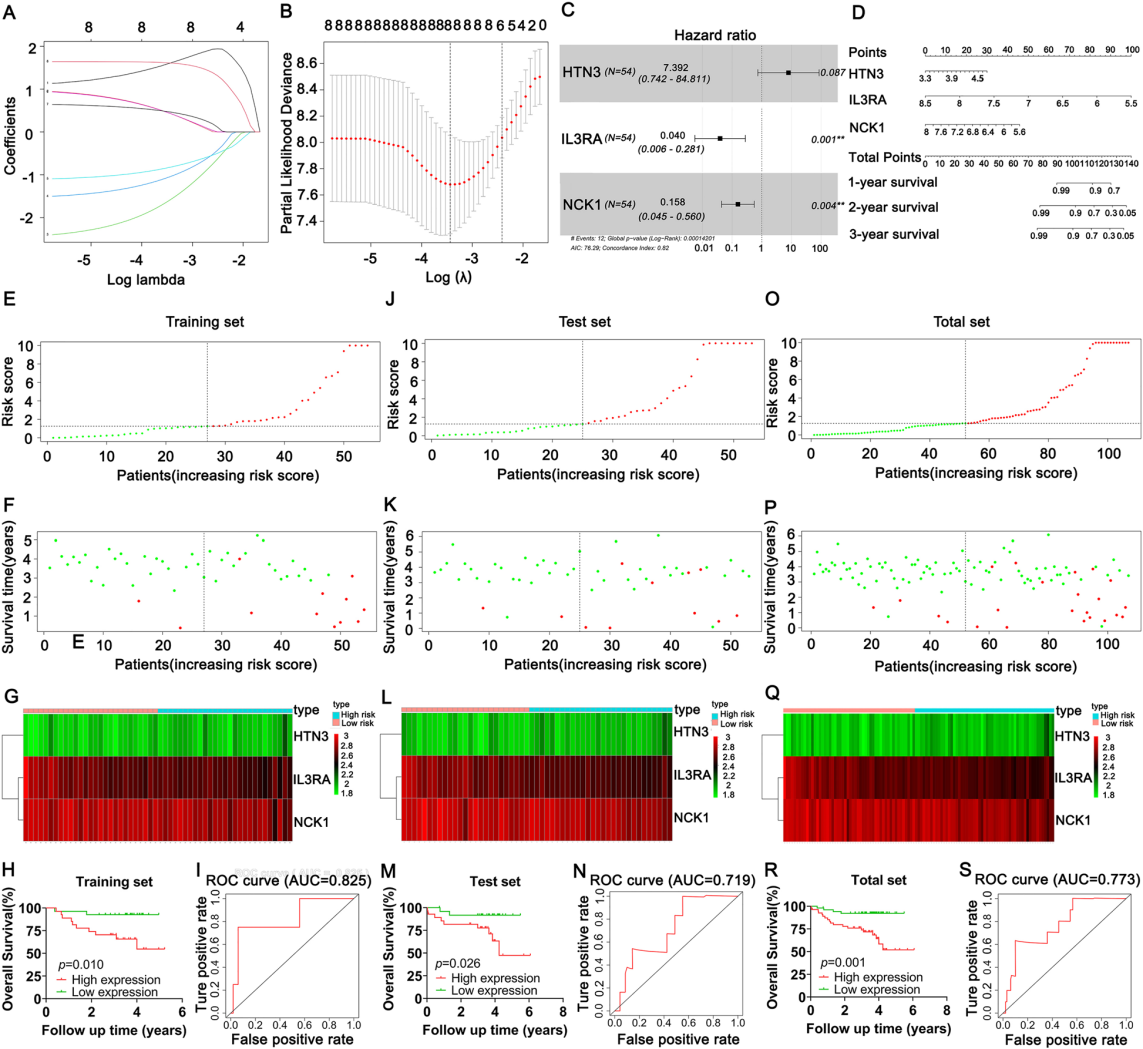
Construction and validation of the novel risk score model. **A-B** The relative regression coefficients of 8 genes identified by the LASSO regression analysis. **C** Multivariate Cox analysis of 3 DEGs. **D** Nomogram of 3 DEGs. **E-I** The distribution of the risk score, survival status, expression of 3 survival-related DEGs in high-risk and low-risk groups, Kaplan–Meier survival curve, ROC curve analyses of the risk score model in the training set. **J-N** The distribution of the risk score, survival status, expression of 3 survival-related DEGs in high-risk and low-risk groups, Kaplan–Meier survival curve, ROC curve analyses of the risk score model in the test set. **O-S** The distribution of the risk score, survival status, expression of 3 survival-related DEGs in high-risk and low-risk groups, Kaplan–Meier survival curve, ROC curve analyses of the risk score model in the total set

In this study, in order to reduce ethnic differences and the limitation of small samples, we studied the relationship between DEGs and OS in the genome microarray map GSE22762 to identify key genes in association with the prognosis of CLL patients. The dataset was divided into two groups: training set and test set. The training set was used for establishing novel risk score model. We used test and total sets to verify the stability and predictive power of the risk score model. According to the univariate Cox regression analysis, 8 genes were obviously in association with OS in the training set (Table 1). To avoid overfitting, we performed LASSO regression to further analyze these 8 survival-related genes (Fig. 2A, B). Multivariate Cox regression finally screened out 3 survival-related genes (HTN3, IL3RA and NCK1) for risk model establishment. Among them, IL3RA and NCK1 were independent predictors (Table 1; Fig. 2 C). Subsequently, we drew a nomogram consisting HTN3, IL3RA and NCK1 to quantitatively predict 1-, 2- and 3-year OS in CLL patients (Fig. 2D). The calculation method of the novel risk score model was as follows: 2.071 * HTN3 expression value + (-3.228) * IL3RA expression value + (-1.847) * NCK1 expression value. The risk score for each CLL patient in the training, test and total sets was calculated according to the above formular. In accordance with the median risk score of training set, we divided CLL patients into low- and high-risk group.

**Table 1:** Univariate and multivariate analyses of overall survival for differentially expressed genes (DEGs) in chronic lymphocytic leukemia (CLL) patients

	**Univariate analysis**		**Multivariate analysis**		
**Genes**	**HR (95%CI)**	***P-*value**	**Coef**	**HR (95%CI)**	***P-*value**
AGFG2	386.110(9.876–15,094.626)	0.001^**^	ND	ND	ND
NCK1	0.232(0.084–0.643)	0.005^**^	-1.847	00.158(0.044–0.560)	0.004^**^
TEKT3	28.499(2.209–367.667)	0.010^*^	ND	ND	ND
IL3RA	0.077(0.011–0.547)	0.010^*^	-3.228	0.040(0.006–0.281)	0.001^**^
SIT1	5.577(1.359–22.893)	0.017^*^	ND	ND	ND
HTN3	10.582(1.172–95.560)	0.036^*^	2.071	7.933(0.742–84.811)	0.087
INIP	0.069(0.005–0.901)	0.041^*^	ND	ND	ND
TCF7L1	10.046(1.011–99.831)	0.049^*^	ND	ND	ND

^*^p < 0.05

^**^p < 0.01

K-M survival curve indicated that OS in the low-risk group was longer than that in high-risk group in the training set (*p* = 0.010; Fig. 2H), test set (*p* = 0.026; Fig. 2M) and total set (*p* = 0.001; Fig. 2R). The effectiveness of the risk score were assessed by the receiver operating characteristic (ROC). The areas under the curve (AUC) of training set, test set and total set were 0.825, 0.719 and 0.773, respectively (Fig. 2I, N, S). The risk scores of CLL patients in the training set, test set and total set were ranked and their distribution were shown in Fig. 2E, J, O. The dot plots demonstrated the CLL patients’ survival status in the training, test and total sets (Fig. 2F, K, P). The expression of 3 survival-related DEGs between low- and high-risk groups were displayed in the heatmap (Fig. 2G, L, Q).

### Immune signatures of risk groups and the correlation between risk groups of CLL patients with cytogenetic abnormality

**Fig. 3:**
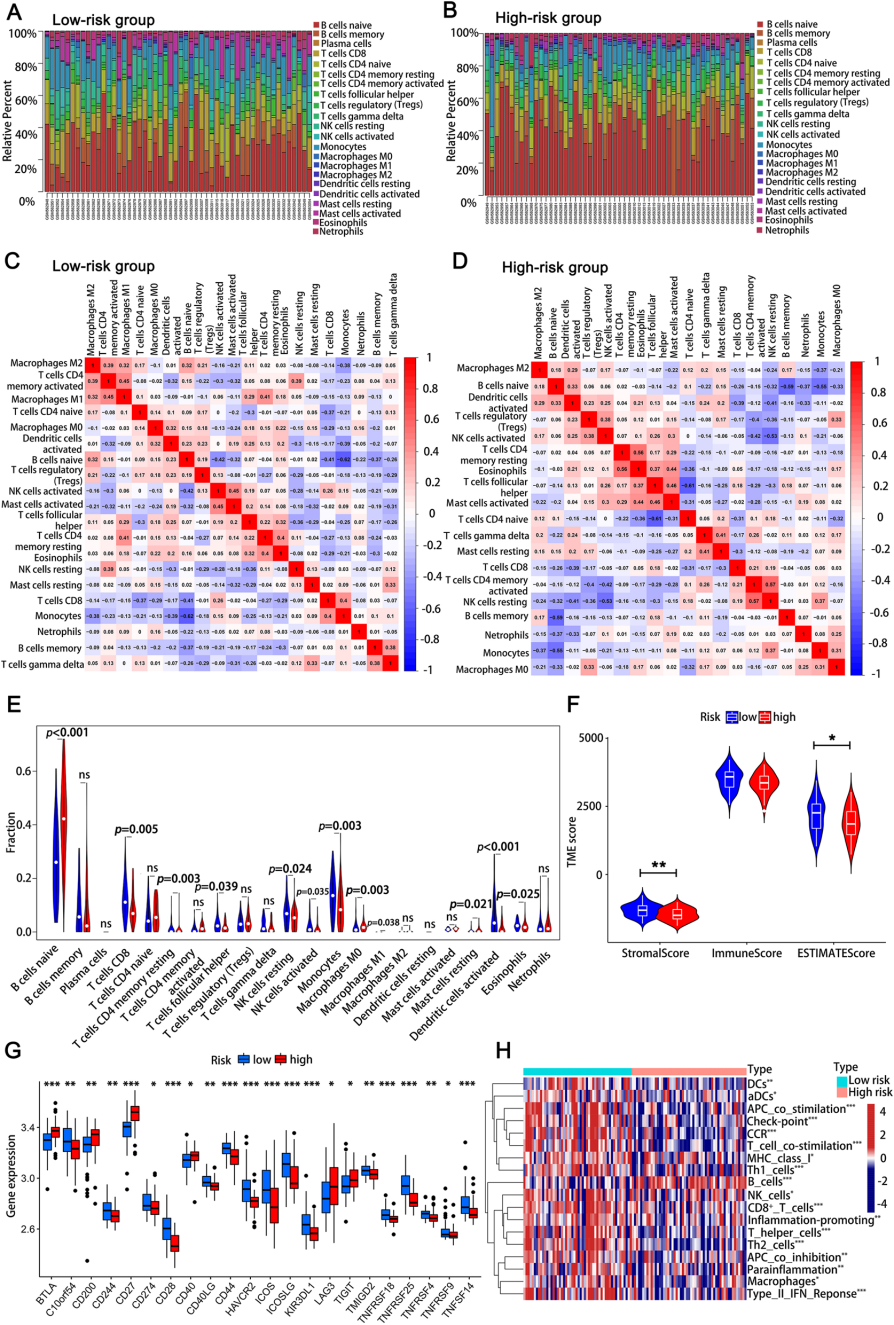
Immune signatures of low- and high-risk groups of CLL patients. **A** The infiltration of 22 types of immune cells in the low-risk group. **B** The infiltration of 22 types of immune cells in the high-risk group. **C** Correlations between immune cells in low-risk group. **D** Correlations between immune cells in high-risk group. **E** Differential expression of immune cells in low- and high-risk groups. **F** The stromal score, immune score and ESTIMATE score in high-risk group were lower than those in low-risk group. **G** Differential expression of immune checkpoint in low- and high-risk groups. **H** The heatmap of immune function analysis in low- and high-risk groups. (^*^p < 0.05, ^**^p < 0.01, ^***^p < 0.001)

We revealed the immune signatures of the low- and high-risk groups. Histogram showed that the proportion of neutrophils in the high-risk group is higher than that in the low-risk group (Fig. 3A, B). The correlation between immune cells in the two groups were shown in (Fig. 3C, D). The vioplot demonstrated the differential expression of 22 reported immune cell types between low- and high-risk groups. B cells naïve (*p* < 0.001), T cells CD8 (*p* = 0.005), CD4 memory resting T cells (*p* = 0.003), follicular helper T cells (*p* = 0.039), resting NK cells (*p* = 0.024), activated NK cells (*p* = 0.035), monocytes (*p* = 0.003), macrophages M0 (*p* = 0.003), macrophages M1 (*p* = 0.038), resting mast cells (*p* = 0.021), activated mast cells (*p* < 0.001) and eosinophils (*p* = 0.025) were obviously associated with risk group (Fig. 3E). The stromal score representing stroma feature of the tumor microenvironment in the high-risk group were lower than that of the low-risk group (*p* < 0.01). The immune score that representing immune cell signatures of high-risk group was lower compared to low-risk group, but there was no statistical significance (Fig. 3F). Moreover, distinct expression of immune checkpoints between the two groups indicated that BTLA (*p* < 0.001), CD200 (*p* < 0.01), CD27 (*p* < 0.001), CD40 (*p* < 0.05), LAG3 (*p* < 0.05) and TIGIT (*p* < 0.05) expression levels were evidently higher in high-risk group (Fig. 3G). Heatmap of immune function analysis demonstrated that the immune status of the high-risk group was more suppressed compared to the low-risk group (Fig. 3H). The effects of different immune functions on OS of CLL patients were shown in Supplementary Fig. 2. We subsequently investigated the relationship between the risk groups and cytogenetic abnormality by chi-square test in GSE25571. The results demonstrated that the high-risk group were related to del 13q14 and del 13q14 single (Table 2).

**Table 2:** Correlation between risk groups of CLL patients and cytogenetic abnormality

	**Patients, No**				
**Characteristics**	**No**	**Low risk**	**High risk**	**χ**^**2**^	***P-*value**
Del 13q14				7.210	0.007^**^
Yes	63	22	41		
No	46	28	18		
Del 13q14 single				3.941	0.047^*^
Yes	46	16	30		
No	63	34	29		
Del 11q22-23				0.854	0.355
Yes	12	4	8		
No	97	46	51		
Del 17p13				1.293	0.255
Yes	9	2	7		
No	100	48	52		

^*^p < 0.05

^**^p < 0.01

### The correlation between DEmiRNAs and clinical characteristics

**Fig. 4:**
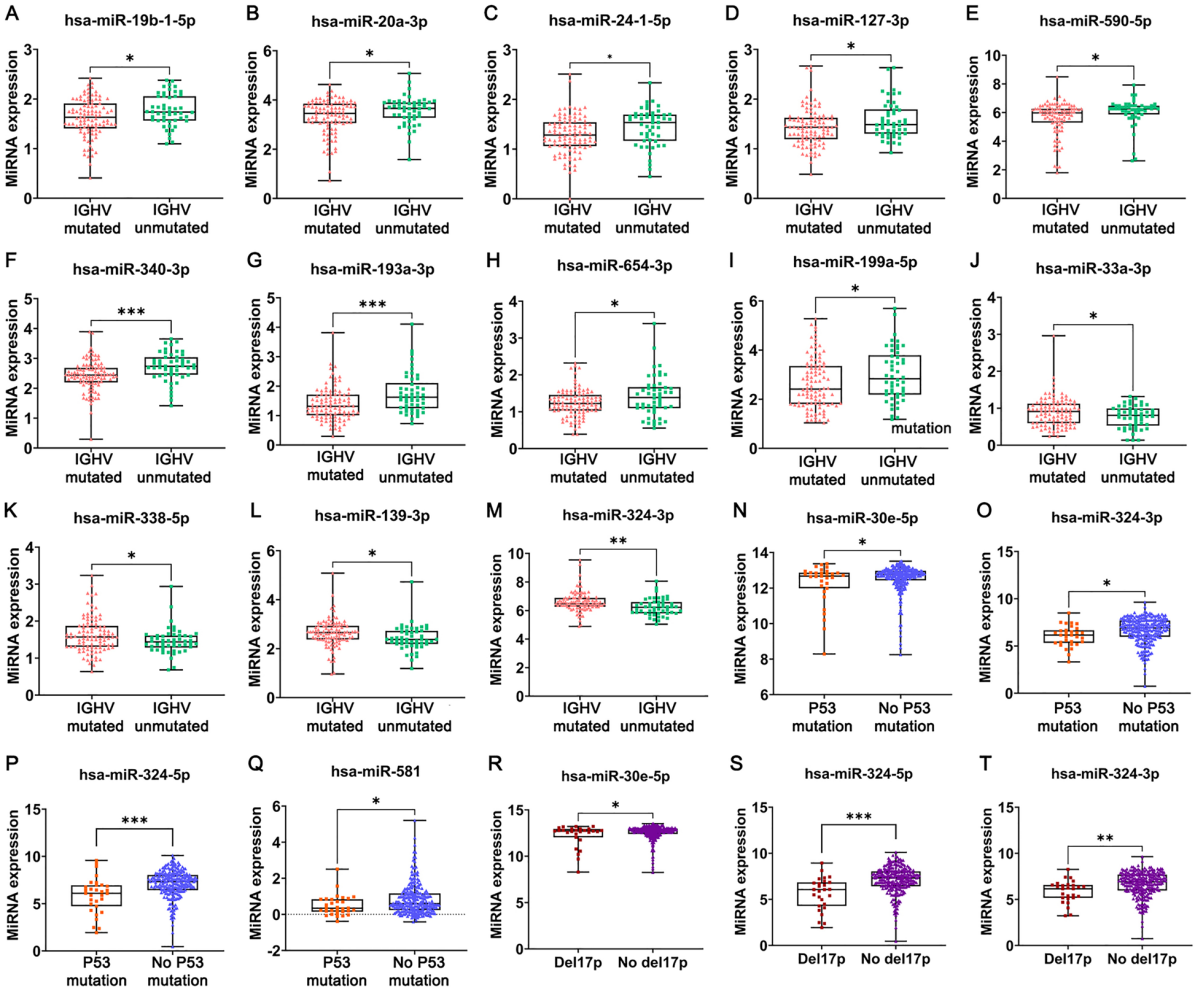
The relationship between differentially expressed miRNAs (DEmiRNAs) and clinical characteristics. **A-M** DEmiRNAs associated with mutational status of IGHV. **N-Q** DEmiRNAs associated with p53 mutational status. **R-T** DEmiRNAs associated with del17p. (^*^p < 0.05, ^**^p < 0.01, ^***^p < 0.001)

To explore the prognostic values of the DEmiRNAs, we analysis the relationship between DEmiRNAs and the mutational status of IGHV, p53 mutational status and del17p in the GSE40533 and GSE45328. In total, we identified 13 DEmiRNAs that related to IGHV mutational status. Among them, the high expression of miR-19b-1-5p (*p* < 0.05), miR-20a-3p (*p* < 0.05), miR-24-1-5p (*p* < 0.05), miR-127-3p (*p* < 0.05), miR-590-5p (*p* < 0.05), miR-340-3p (*p* < 0.001), miR-193a-3p (*p* < 0.001), miR-654-3p (*p* < 0.05) and miR-199a-5p (*p* < 0.05) were associated with IGHV unmutated status (Fig. 4A-I), at the same time, the high expression of miR-33a-3p (*p* < 0.05), miR-338-5p (*p* < 0.05), miR-139-3p (*p* < 0.05) and miR-324-3p (*p* < 0.01) were related to IGHV gene mutation (Fig. 4J-M). Additionally, the low expression of miR-30e-5p (*p* < 0.05), miR-324-3p (*p* < 0.05), miR-324-5p(*p* < 0.001) and miR-581 (*p* < 0.05) were related to p53 mutation (Fig. 4N-Q). We also demonstrated that the low expression of miR-30e-5p (*p* < 0.05), miR-324-5p (*p* < 0.001) and miR-324-3p (*p* < 0.01) were associated with del17p (Fig. 4R-T).

### Prediction and construction of the ceRNA networks

**Fig. 5:**
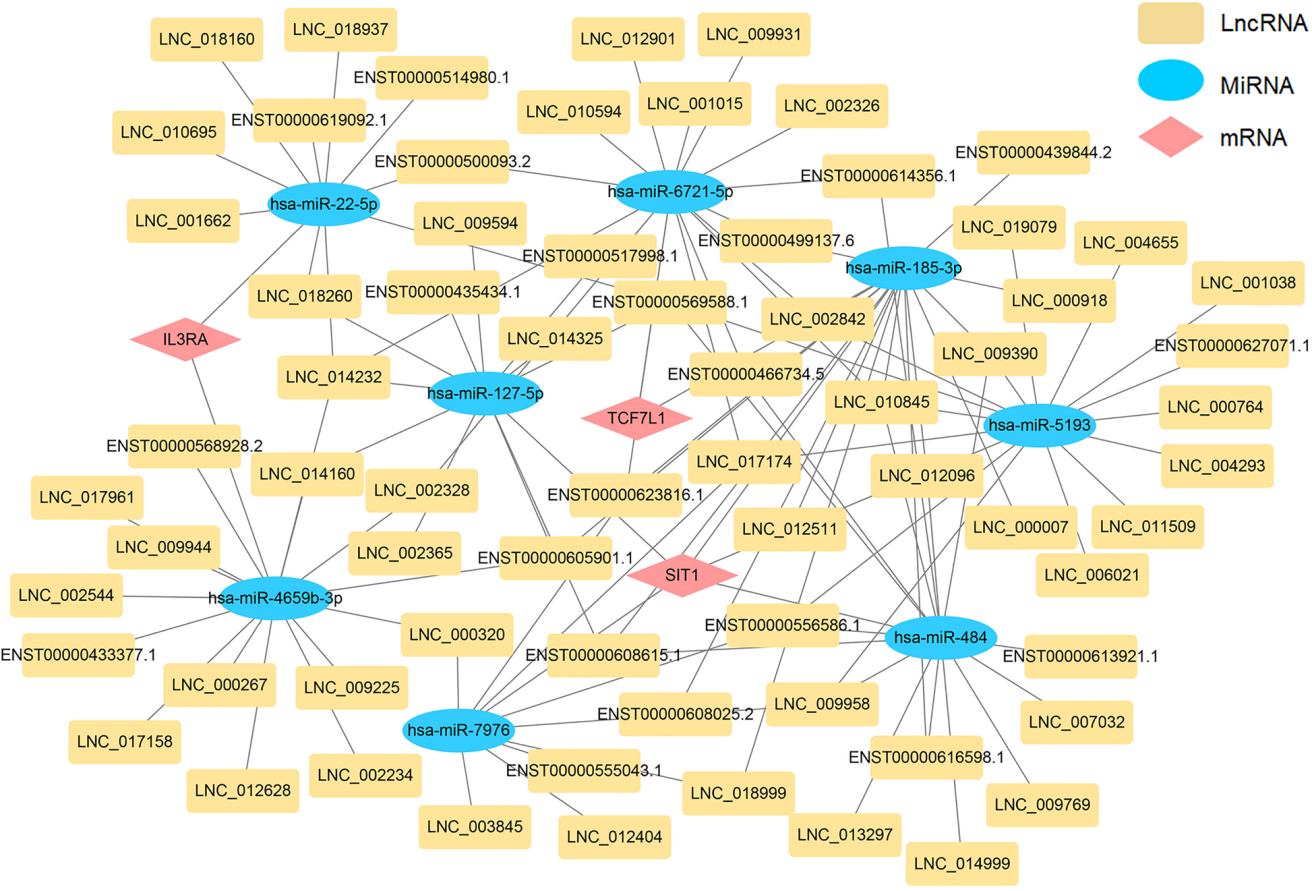
The lncRNA-miRNA-mRNA competing endogenous RNA (ceRNA) network between CLL patients and healthy donors

**Fig. 6:**
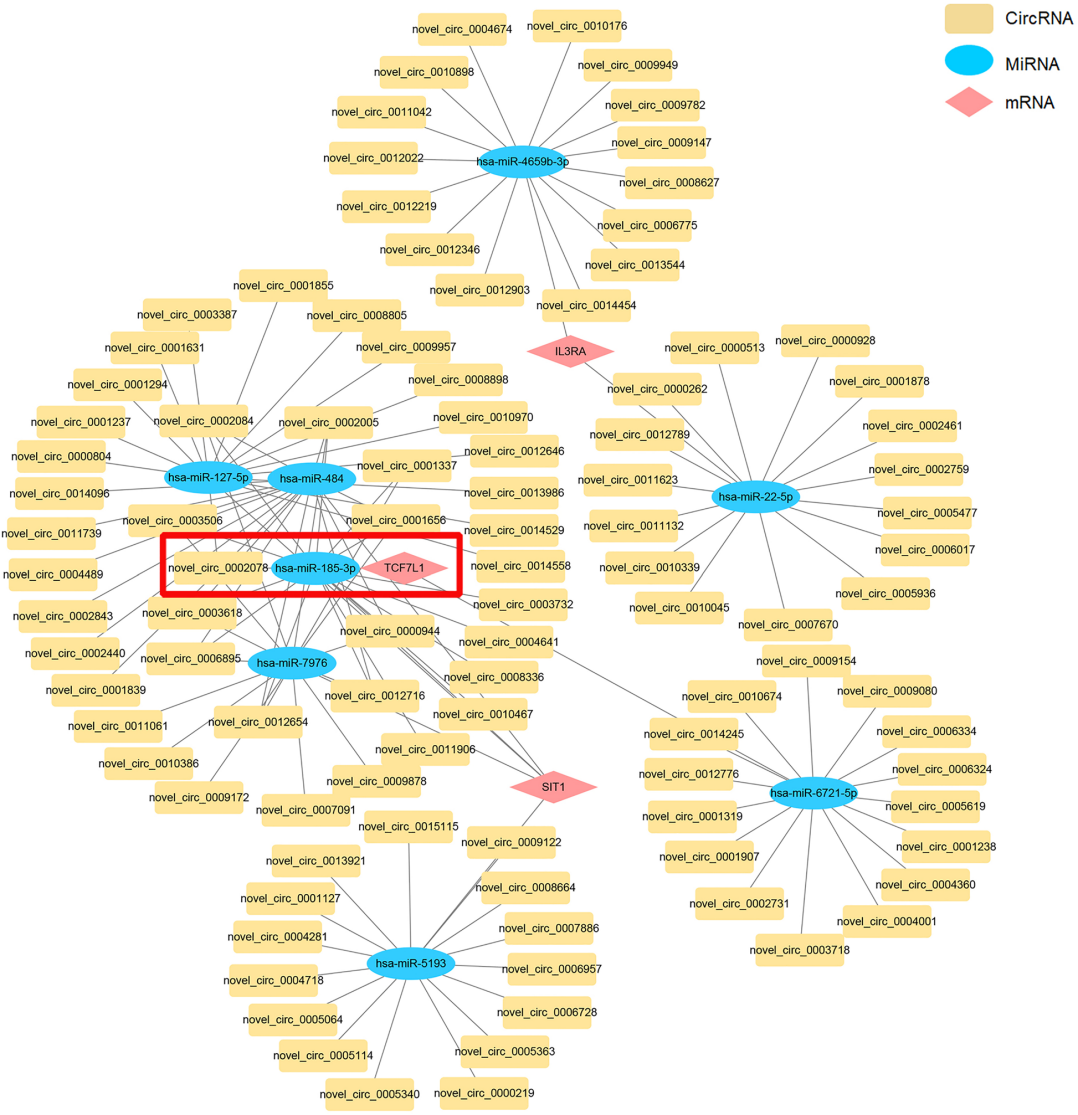
The circRNA-miRNA-mRNA ceRNA network between CLL patients and healthy donors

We constructed the lncRNA-miRNA-mRNA ceRNA network according to the survival-related genes, DEmiRNAs and DElncRNA in CLL patients and healthy donors. Totally, 68 lncRNA nodes, 8 miRNA nodes and 3 mRNA nodes were selected to construct the ceRNA network (Fig. 5). To decipher the functions of DEcircRNAs in CLL patients, we constructed an abnormal circRNA‐miRNA‐mRNA ceRNA network. In total, 3 DEmRNAs, 8 DEmiRNAs and 101 DEcircRNAs were included in the ceRNA network (Fig. 6).

### Validation of the circ_0002078/miR-185-3p/TCF7L1 axis

We verified the expression of three DEGs (TRIM34, SLC30A10, HOXD4) and circ_0007675 by qRT-PCR. The relative expression of three DEGs and circ_0007675 in CLL patients were remarkably up-regulated compared with normal B cells (*p* < 0.05; Supplementary Fig. 3A-D).

**Fig. 7:**
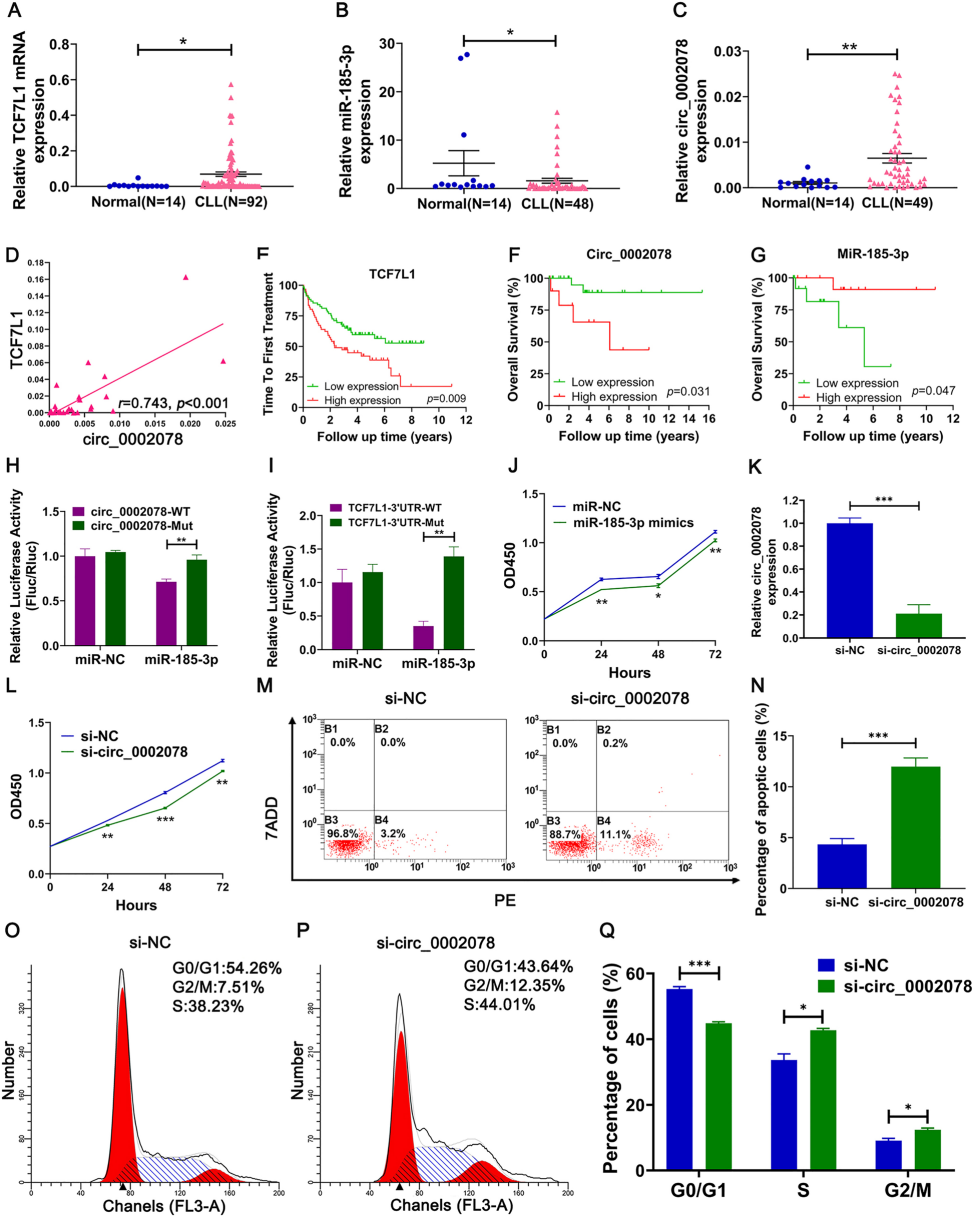
The verification of circ_0002078/miR-185-3p/TCF7L1 axis and the function of circ_0002078/miR-185-3p. **A** The expression of TCF7L1 in patient specimens were significantly increased. **B** The expression of miR-185-3p in patient specimens were significantly decreased. **C** The expression of circ_0002078 in patient specimens were significantly increased. **D** There was a strong linear correlation between TCF7L1 and circ_0002078. **E** Higher expression of TCF7L1 was associated with shorter TTFT. **F** Lower expression of miR-185-3p was associated with shorter OS. **G** Higher expression of circ_0002078 was associated with shorter OS. **H** The luciferase activity analysis showed that miR-185-3p can bind to the circ_0002078. **I** The luciferase activity analysis shows that miR-185-3p can bind to 3′UTR of TCF7L1. **J** Overexpression of miR-185-3p inhibited cell proliferation. **K** Efficiency verification of circ_0002078 knockdown in EHEB cells by qRT-PCR. **L** Knockdown of circ_0002078 inhibited cell proliferation. **M**, **N** Knockdown of circ_0002078 promoted cell apoptosis. **O-Q** Knockdown of circ_0002078 led to G2/M cell cycle arrest. All the results are expressed as mean ± SEM. (^*^p < 0.05, ^**^p < 0.01, ^***^p < 0.001)

The expression of TCF7L1, miR-185-3p, and circ_0002078 in the CLL patients and healthy donors were detected respectively. The results showed that TCF7L1 as well as circ_0002078 were up-regulated (*p* < 0.05; Fig. 7A and C), whereas miR-185-3p was down-regulated in CLL patients compared to normal B cells (*p* < 0.05; Fig. 7B). Pearson correlation analysis revealed the strong linear correlation between TCF7L1 and circ_0002078 (*r* = 0.743, *p* < 0.001; Fig. 7D). K-M survival analysis in GSE39671 demonstrated that higher expression of TCF7L1 was correlated to shorter time to first treatment (TTFT) (*p* = 0.009; Fig. 7E). Based on the clinical information and expression levels in CLL patients, we performed the survival analysis of circ_0002078 and miR-185-3p. The high expression of circ_0002078 was associated with a shorter OS (*p* = 0.031; Fig. 7F). CLL patients with higher miR-185-3p expression had longer OS than patients with lower expression of miR-185-3p (*p* = 0.047; Fig. 7G).

The interrelationship among circ_0002078, miR-185-3p and TCF7L1 were detected by dual-luciferase reporter gene assay. When luciferase reporter plasmid containing circ_0002078-WT (or TCF7L1-WT) co-transfected with miR-185-3p mimics into 293 T cells, the luciferase activities were evidently decreased compared to that co-transfected with miR-NC. However, when the luciferase reporter plasmid containing circ_0002078-Mut (or TCF7L1-Mut) co-transfected with miR-185-3p mimics and miR-NC respectively into 293 T cells, there was no significant difference between the results of two groups (*p* < 0.01; Fig. 7H, I).

To explore the role of miR-185-3p on cell proliferation, we performed CCK-8 assay. We transferred miR-185-3p mimics and miR-NC into MEC1 cells. The expression of miR-185-3p was up-regulated in MEC1 cells (*p* < 0.01; Supplementary Fig. 4A). The OD values of miR-185-3p overexpression group were significantly lower than control group (Fig. 7J), which indicated that miR-185-3p inhibited cell proliferation of the CLL cells. We predicted the potential functions of target genes of circ_0002078 by GO and KEGG analyses. GO analysis results demonstrated that the target genes of circ_0002078 were enriched in positive regulation of gene expression, lymphocyte proliferation and regulation of B cell apoptotic process (Supplementary Fig. 4B). KEGG pathway analysis showed that the target genes were primarily relevant to JAK-STAT signaling pathway and cytokine-cytokine receptor interaction (Supplementary Fig. 4C). The results suggested that circ_0002078 may promote the occurrence and development of CLL in terms of cell proliferation and apoptosis. We transfected siRNA of circ_0002078 in EHEB cells and the expression of circ_0002078 was down-regulated (*p* < 0.001; Fig. 7K). CCK-8 assay and apoptosis analysis showed that the knockdown of circ_0002078 obviously inhibited cell proliferation (Fig. 7L) and promoted cell apoptosis (*p* < 0.001; Fig. 7M, N). EHEB cells were blocked in G2/M phase after the transfection of si-circ_0002078 (Fig. 7O-Q). These results suggested that targeting circ_0002078 is a promising direction for the future treatment of CLL.

**Fig. 8:**
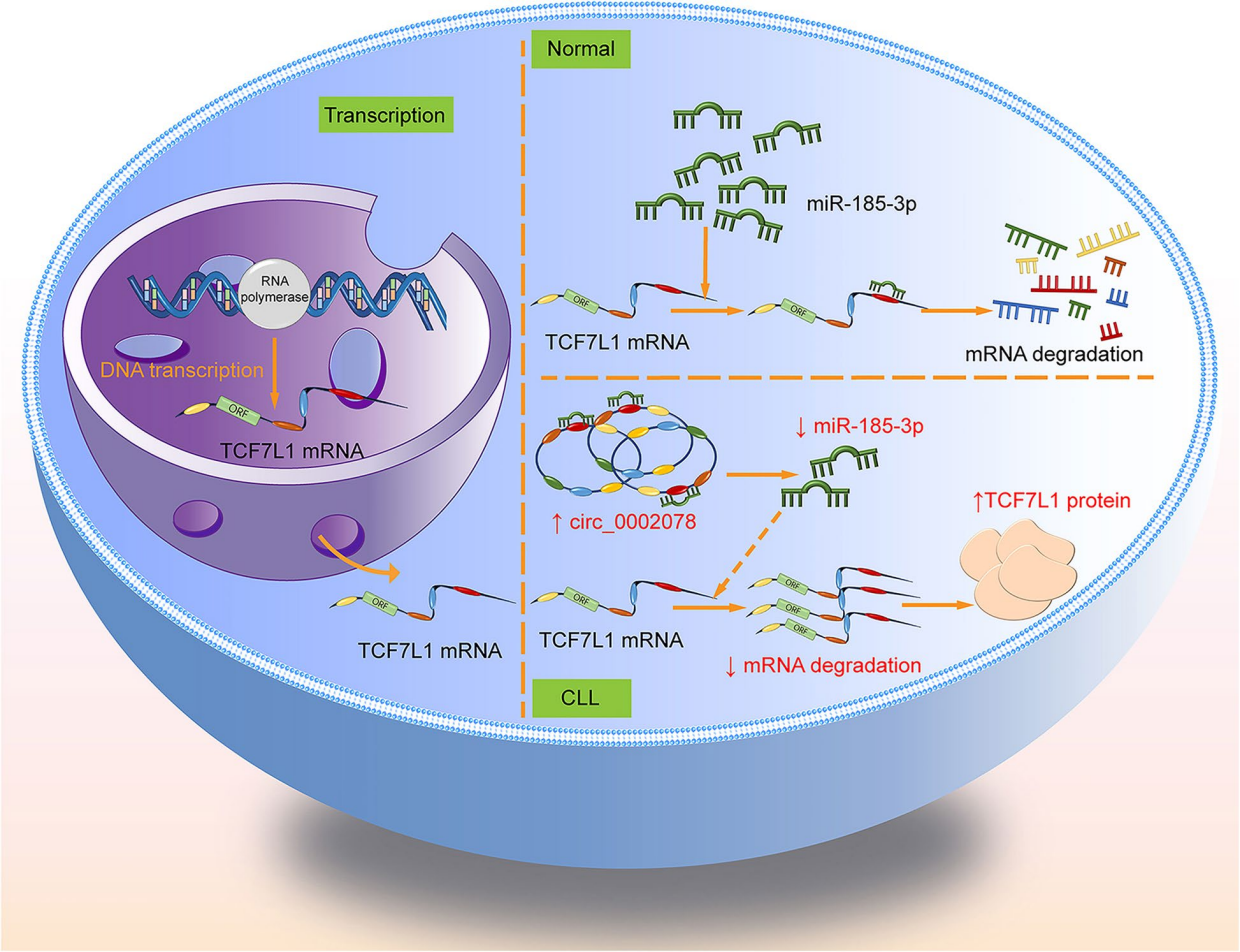
The mechanism of circRNA circ_0002078/miR-185-3p/TCF7L1 axis in CLL cells. The interaction mechanism between them is that circ_0002078 can absorb miR-185-3p like a sponge, thereby inhibiting the inhibitory effect of miR-185-3p on TCF7L1. This competitive combination leads to the increasing expression of TCF7L1, which contributes to the promotion of cell proliferation, metastasis and other life processes that can conducive to the development of cancer

The interaction mechanism among circ_0002078, miR-185-3p and TCF7L1 is that circ_0002078 can absorb miR-185-3p like a sponge, accordingly suppressing the repression of miR-185-3p on TCF7L1. In CLL, the expression of circ_0002078 is abnormally elevated, therefore the binding of miR-185-3p to TCF7L1 is correspondingly reduced, leading to the decrease of the TCF7L1 mRNA degradation, and eventually resulting in the up-regulation of TCF7L1, which contributes to the promotion of cell proliferation, metastasis and other life processes that can conducive to the development of CLL (Fig. 8).

## Discussion

In the present study, we analyzed ceRNA networks obtained from RNA sequencing in CLL patients and cell lines for the first time. The potential biological functions and prognostic prediction value of the DEGs were explored. A novel risk score model composed of HTN3, IL3RA and NCK1 was established to facilitate prognostic assessment. MiR-324-3p was identified in association with IGHV unmutated status, p53 mutation and del17p. The survival-related ceRNA networks provided fundamental clues for promising prognostic biomarkers and therapeutic vulnerabilities of CLL. Moreover, the validation of circ_0002078/miR-185-3p/TCF7L1 axis provided pioneering insights into the molecular underpinnings of CLL.

CLL is a B-cell malignancy occurs mainly in advanced age and have changeable response to treatments [26, 27]. CLL presents a variable disease course and typically involves chromosomal deletions [28, 29]. The age of onset, genetic profile and other characteristics between different races are heterogenous [30]. Due to the RNA sequencing technology, a plethora of ncRNAs have been explored and confirmed to exert pivotal roles in different physiopathological processes of cells [31, 32]. CeRNA network is a crucial mechanism in post-transcriptional layer of gene translation regulation [33]. These ceRNAs contain many kinds of RNAs, such as circRNAs, lncRNAs and pseudogenes, and they regulate gene expression by competing for the same MREs [34]. Recently, accumulating studies have showed that the lncRNA/circRNA-miRNA-mRNA ceRNA networks played crucial roles in tumor onset and development [35, 36]. However, the roles of ceRNA networks in CLL remain uncovered. In the present investigation, we identified the DEmRNAs, DEmiRNAs, DElncRNAs and DEcircRNAs between CLL patients and healthy donors as well as CLL cells and normal controls. GO analysis results demonstrated that these DEGs between CLL specimens and control were significantly related to sequence-specific DNA binding, chromosomal part and cellular biosynthetic process. KEGG analysis indicated that these DEGs enriched in pathways that were correlated with oncogenesis and tumor development. Notch signaling is deregulated in multiple solid tumors and hematological malignancies [37]. JAK-STAT signaling pathway played important roles in driving aggressive growth, invasion, therapeutic resistance and tumor-mediated immunosuppression [38]. These analyses indicated that the identified DEGs are correlated with the tumorigenesis and progression of CLL, which is worthy of our further exploration.

Understanding of prognostic biomarkers and risk score systems contributes to the prognosis of CLL [39]. In this study, we identified 8 DEGs that associated with the OS of CLL via survival analysis. Among them, IL3RA and NCK1 were independent prognostic indicators for the OS in CLL patients. Subsequently, we constructed a novel risk score model that showed highly robust predictive power for OS. The high-risk group was associated with del(13q14) as well as shorter OS. Distinct immune signatures in two groups indicated that the proportion of macrophages and activated CD4^+^ memory T cells were larger in high-risk group. Yucai Wang et al. discovered that Richter syndrome nodal tissue had higher infiltration of FOXP3^+^ T cells and CD163^+^ macrophages [40]. The activated CLL signature was obviously related to macrophages M2 and activated CD4^+^ memory T cells [41]. Collectively, these findings implied the intrinsic relations between the risk score model and immune pathways, which provide valuable clues for promising drug discovery in further investigations.

Recently, studies of miRNAs as biomarkers for diagnosis and treatment of disease have attracted great attention. For example, circulating microRNAs are important predictors of fasting blood glucose in prediabetic patients [42]. MicroRNA let-7b downregulates the expression of oncogene AML1-ETO, making it a potential therapeutic target in t (8;21) AML [43]. MiR-142-5p can differentiate between treatment-naïve CML patients who did not respond to imatinib during treatment and those who did [44]. Nevertheless, the roles of miRNA in CLL are largely unexplored. The current study identified DEmiRNAs that related to IGHV mutational status, p53 mutational status and del17p. Intriguingly, the low expression of miR-324-3p was associated with IGHV unmutated status, p53 mutation and del17p, which implied that low expression of miR-324-3p was related to dismal prognosis. The potential functions of miR-324-3p in CLL deserve further discussion.

Based on the influence of DEGs on prognosis and DElncRNA/DEcircRNA in CLL patients, we established the lncRNA-miRNA-mRNA and circRNA-miRNA-mRNA networks. These intricate and intertwined networks contributed to uncovering the regulatory mechanism of the ceRNAs in CLL. The key nodes in the networks presented an obvious relevance to OS, which imply that they play significant roles in survival prediction. Among the DEmiRNAs in our RNA-sequencing data, miR-185-3p was found to be downregulated and associated with chemosensitivity [45, 46] and cell proliferation inhibition [47]. In addition, miR-185-3p is involved in cancer progression as a key molecule in the ceRNA networks [48–50]. In CLL, the mechanisms of drug resistance and cell proliferation have not been fully elucidated and the potential role of miR-185-3p in CLL deserves further investigation. Study findings of predicted target genes regulated by miR-185-3p indicated that TCF7L1 is highly expressed in prostate cancer [51], skin squamous cell carcinoma [52] and gastric cancer [53] and is related to their occurrence and progression. The regulation of TCF7L1 by miR-185-3p may be one of its potential mechanisms to promote tumorigenesis and development in CLL. Circ_0002078 was screened by intersecting the predicted miR-185-3p-binding circRNAs with the differentially expressed circRNAs in our RNA-sequencing results.

Therefore, in the current investigation, we verified circ_0002078/miR-185-3p/TCF7L1 axis through qRT-PCR, explored their functions and regulatory relationship. The expression of miR-185-3p was down-regulated in CLL patients and its overexpression inhibited the proliferation of MEC1 cells. The expression and function of circ_0002078 were explored in CLL patients and cell lines. The isoforms of circ_0002078 have not been reported in the existing studies, so we did not detect the isoforms of circ_0002078. The overexpression of circ_0002078 in CLL and its role in promoting proliferation, inhibiting cell apoptosis and increasing cell cycle progression make it a promising target for the treatment of CLL. The dual-luciferase reporter gene assay results indicated that miR-185-3p targeted circ_0002078 and 3’UTR of TCF7L1 at the MREs we predicted. In short, these results indicated the potential role of circ_0002078/miR-185-3p/TCF7L1 aixs in CLL.

## Conclusion

In summary, we identified the distinct expression profile of lncRNAs, circRNAs, miRNAs and mRNAs in CLL specimens and CLL cells. Functional enrichment analyses uncovered the biological functions of DEGs. Subsequently, we constructed a risk score model consisting of HTN3, IL3RA and NCK1 to predict OS in CLL. The distinct immune signatures in low- and high-risk groups provided new insight into the exploration of immune molecular underpinnings and immunotherapeutic targets discovery. In addition, we constructed ceRNA networks associated with prognosis. The current study is helpful in enriching deeper understanding of the molecular mechanism in CLL pathogenesis and providing directions for exploring novel prognostic biomarkers.

## Supplementary Information

**Additional file 1: Supplementary figure 1.** Functional enrichment analyses of differentially expressed genes (DEGs) in chronic lymphocytic leukemia (CLL) cell lines. (A) GO analysis results showed that changes in MF, CC and BP of DEGs between CLL cell lines and control were mainly enriched in sequence−specific DNA binding, Golgi membrane and cation transmembrane transport. (B) KEGG enrichment analysis of DEGs in CLL cell lines and control were mainly enriched in mTOR signaling pathway, NF-kappaB signaling pathway and cell cycle.

**Additional file 2: Supplementary figure 2.** The relationship between immune-related function and prognosis of CLL patients.

**Additional file 3: Supplementary figure 3.** The expression of TRIM34, SLC30A10, HOXD4 and circ_0007675 in CLL patients and normal B cells.(A) The expression of TRIM34 in patient specimens were significantly increased. (B) The expression of SLC30A10 in patient specimens were significantly increased. (C) The expression of HOXD4 in patient specimens were significantly increased. (D) The expression of circ_0007675 in patient specimens were significantly increased. All results are expressed as mean ± SEM.

**Additional file 4: Supplementary figure 4.** Efficiency of miR-185-3p transfection and functional enrichment analyses of target genes of circ_0002078. (A) Efficiency verification of miR-185-3p overexpression in MEC-1 cells by qRT-PCR. (B) GO analysis results demonstrated that the target genes of circ_0002078 were enriched in positive regulation of gene expression, lymphocyte proliferation, and regulation of B cell apoptotic process. (C) KEGG pathway analysis showed that the target genes were primarily relevant to JAK-STAT signaling pathway and cytokine-cytokine receptor interaction. 

**Additional file 5: Table S1.** The top 10 up-regulation and down-regulation differentially expressed mRNAs (DEmRNAs) between chronic lymphocytic leukemia (CLL) patients and normal B cells. **Table S2.** The top 10 up-regulation and down-regulation differentially expressed microRNAs (DEmiRNAs) between CLL patients and normal B cells. **Table S3.** The top 10 up-regulation and down-regulation differentially expressed long noncoding RNAs (DElncRNAs) between CLL patients and normal B cells. **Table S4.** The top 10 up-regulation and down-regulation differentially expressed circular RNAs (DEcircRNAs) between CLL patients and normal B cells. **Table S5.** The top 10 up-regulation and down-regulation DEmRNAs between CLL cells and normal B cells. **Table S6.** The top 10 up-regulation and down-regulation DEmiRNAs between CLL cells and normal B cells. **Table S7.** The top 10 up-regulation and down-regulation DElncRNAs between CLL cells and normal B cells. **Table S8.** The top 10 up-regulation and down-regulation DEcircRNAs between CLL cells and normal B cells. **Table S9.** Primer sequences of the genes tested by qRT-PCR.

## Data Availability

Not applicable.

## Abbreviations

CLL: Chronic lymphocytic leukemia
ceRNAs: Competing endogenous RNAs
miRNAs: MicroRNAs
DEmiRNAs: Differentially expressed miRNAs
DEmRNAs: Differentially expressed mRNAs
ncRNAs: Non-coding RNAs
lncRNAs: Long noncoding RNAs
circRNAs: Circular RNAs
MREs: MiRNA response elements
DEGs: Differentially expressed genes
OS: Overall survival
DElncRNAs: Differentially expressed lncRNAs
DEcircRNAs: Differentially expressed circRNAs
GO: Gene Ontology
KEGG: Kyoto Encyclopedia of Genes and Genomes
MF: Molecular function
CC: Cell component
BP: Biological process
FBS: Fetal bovine serum
qRT-PCR: Quantitative real-time polymerase chain reaction
CCK-8: Cell Counting Kit-8
K-M: Kaplan-Meier
ROC: Receiver operating characteristic
AUC: Area under the curve
TTFT: Time to first treatment
