# Supplementary figures and images for: Competing endogenous RNA networks related to prognosis in chronic lymphocytic leukemia: comprehensive analyses and construction of a novel risk score model
Source: Biomark Res. 2022 Oct 21;10:75. doi: 10.1186/s40364-022-00423-y (PMC9585723; doi:10.1186/s40364-022-00423-y)

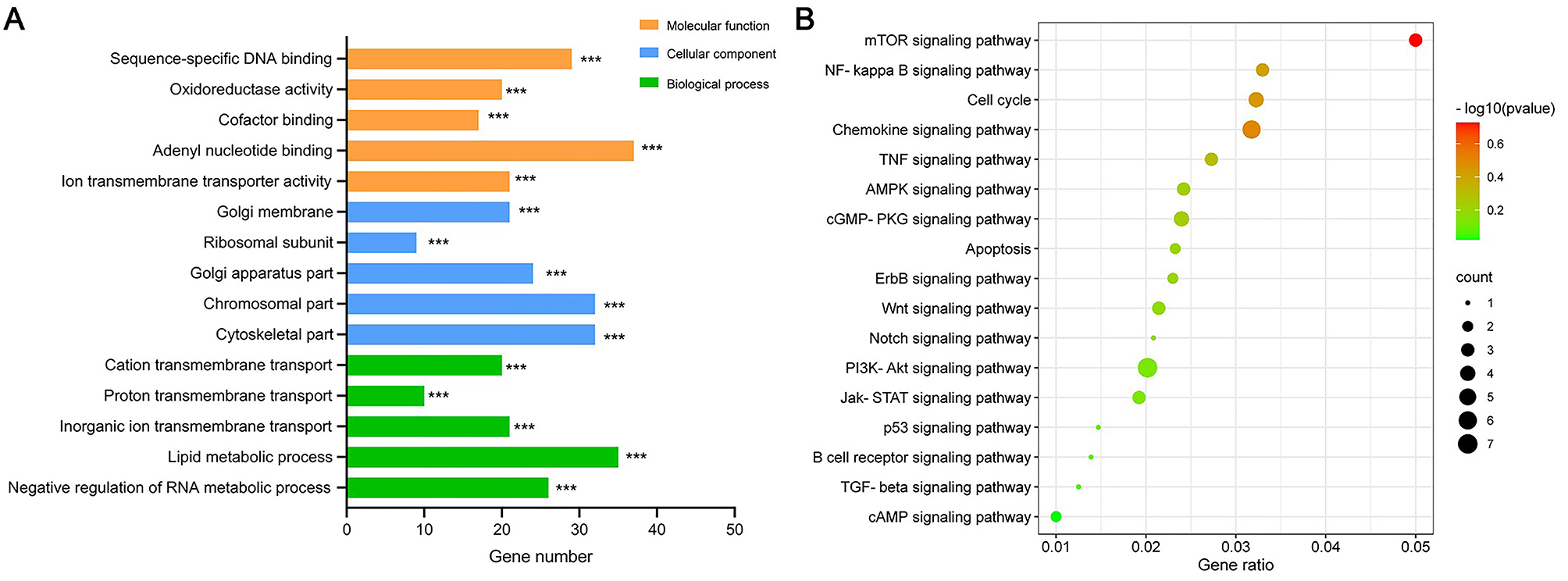

Supplement: Supplementary file 1 — Additional file 1: Supplementary figure 1. Functional enrichment analyses of differentially expressed genes (DEGs) in chronic lymphocytic leukemia (CLL) cell lines. (A) GO analysis results showed that changes in MF, CC and BP of DEGs between CLL cell lines and control were mainly enriched in sequence−specific DNA binding, Golgi membrane and cation transmembrane transport. (B) KEGG enrichment analysis of DEGs in CLL cell lines and control were mainly enriched in mTOR signaling pathway, NF-kappaB signaling pathway and cell cycle. [file 40364_2022_423_MOESM1_ESM.jpg]

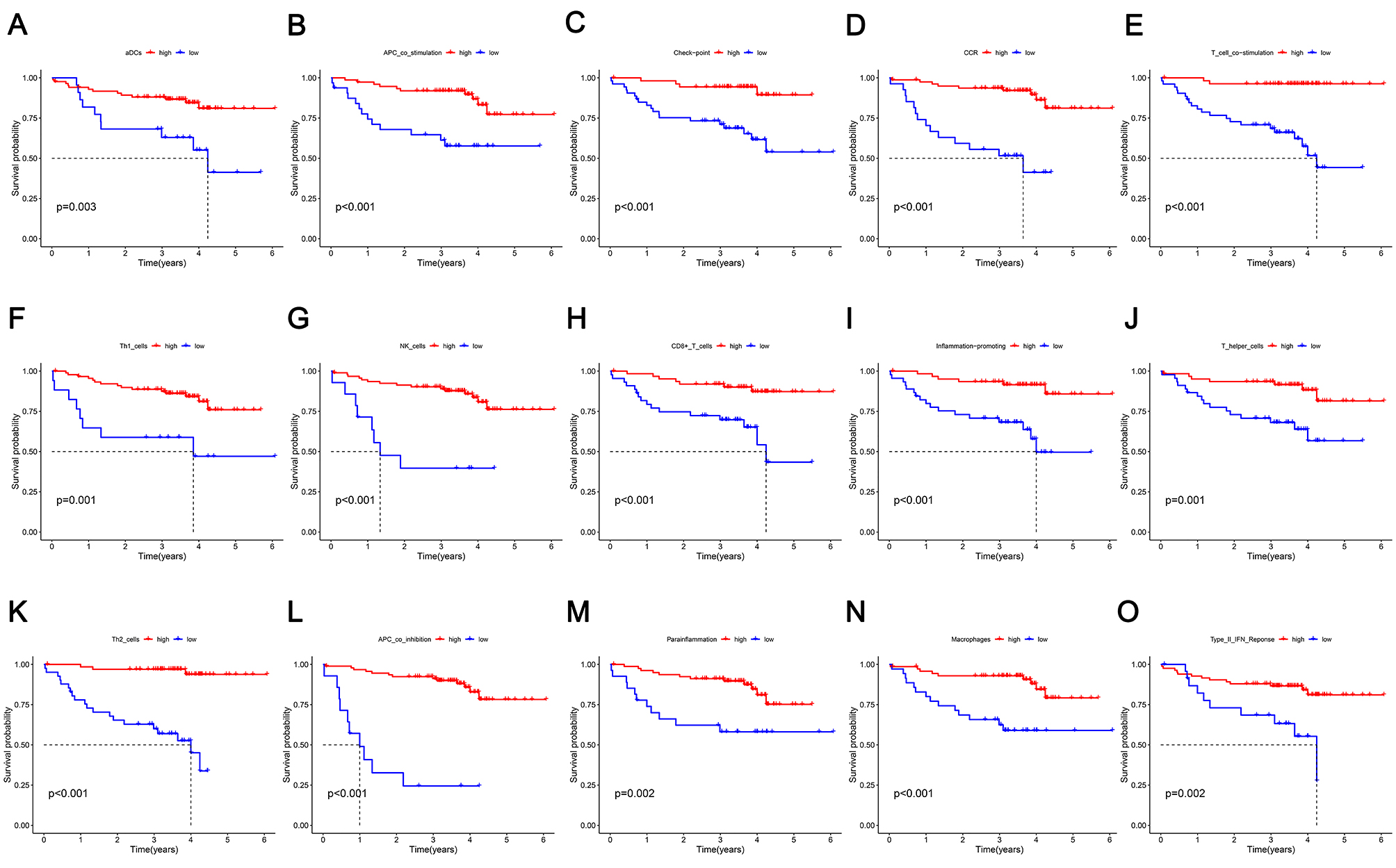

Supplement: Supplementary file 2 — Additional file 2: Supplementary figure 2. The relationship between immune-related function and prognosis of CLL patients. [file 40364_2022_423_MOESM2_ESM.jpg]

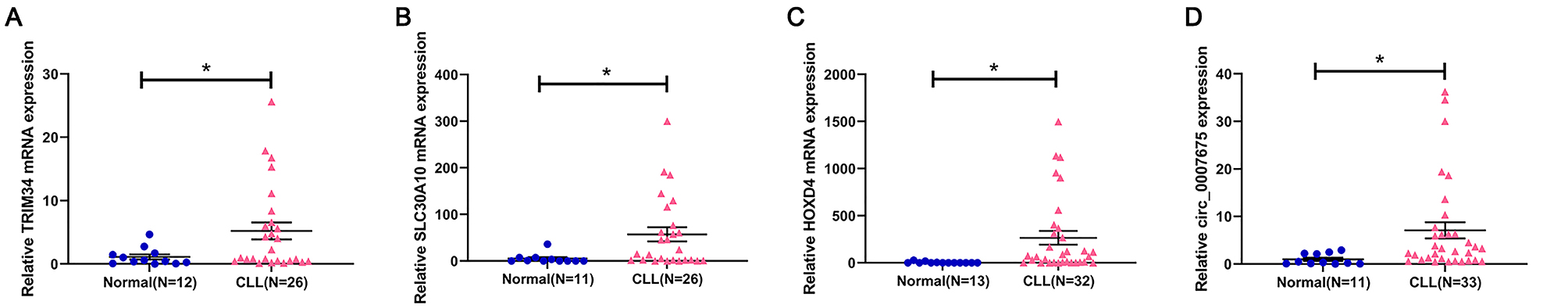

Supplement: Supplementary file 3 — Additional file 3: Supplementary figure 3. The expression of TRIM34, SLC30A10, HOXD4 and circ_0007675 in CLL patients and normal B cells.(A) The expression of TRIM34 in patient specimens were significantly increased. (B) The expression of SLC30A10 in patient specimens were significantly increased. (C) The expression of HOXD4 in patient specimens were significantly increased. (D) The expression of circ_0007675 in patient specimens were significantly increased. All results are expressed as mean ± SEM. [file 40364_2022_423_MOESM3_ESM.jpg]

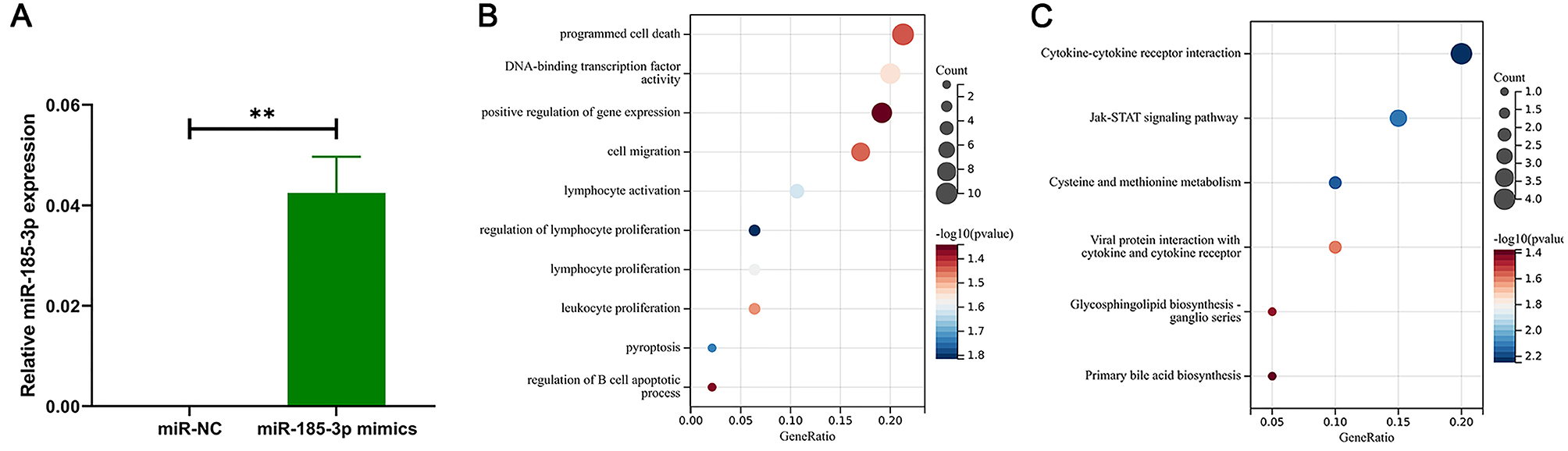

Supplement: Supplementary file 4 — Additional file 4: Supplementary figure 4. Efficiency of miR-185-3p transfection and functional enrichment analyses of target genes of circ_0002078. (A) Efficiency verification of miR-185-3p overexpression in MEC-1 cells by qRT-PCR. (B) GO analysis results demonstrated that the target genes of circ_0002078 were enriched in positive regulation of gene expression, lymphocyte proliferation, and regulation of B cell apoptotic process. (C) KEGG pathway analysis showed that the target genes were primarily relevant to JAK-STAT signaling pathway and cytokine-cytokine receptor interaction. [file 40364_2022_423_MOESM4_ESM.jpg]
