# Supplementary material for: Competing endogenous RNA networks related to prognosis in chronic lymphocytic leukemia: comprehensive analyses and construction of a novel risk score model
Source: Biomark Res. 2022 Oct 21;10:75. doi: 10.1186/s40364-022-00423-y (PMC9585723; doi:10.1186/s40364-022-00423-y)
Supplement: Supplementary file 5 — Additional file 5: Table S1. The top 10 up-regulation and down-regulation differentially expressed mRNAs (DEmRNAs) between chronic lymphocytic leukemia (CLL) patients and normal B cells. Table S2. The top 10 up-regulation and down-regulation differentially expressed microRNAs (DEmiRNAs) between CLL patients and normal B cells. Table S3. The top 10 up-regulation and down-regulation differentially expressed long noncoding RNAs (DElncRNAs) between CLL patients and normal B cells. Table S4. The top 10 up-regulation and down-regulation differentially expressed circular RNAs (DEcircRNAs) between CLL patients and normal B cells. Table S5. The top 10 up-regulation and down-regulation DEmRNAs between CLL cells and normal B cells. Table S6. The top 10 up-regulation and down-regulation DEmiRNAs between CLL cells and normal B cells. Table S7. The top 10 up-regulation and down-regulation DElncRNAs between CLL cells and normal B cells. Table S8. The top 10 up-regulation and down-regulation DEcircRNAs between CLL cells and normal B cells. Table S9. Primer sequences of the genes tested by qRT-PCR. [file 40364_2022_423_MOESM5_ESM.docx]

**Table S1. The top 10 up-regulation and down-regulation differentially expressed mRNAs (DEmRNAs) between chronic lymphocytic leukemia (CLL) patients and normal B cells**

| mRNAs(up-regulation) | foldchange | *p*-value | mRNAs(down-regulation) | foldchange | *p*-value |
| --- | --- | --- | --- | --- | --- |
| ADRB1 | 10.09260897 | 3.16E-07 | HNRNPC | -6.44237 | 0.000471 |
| IL3RA | 8.917241981 | 1.10E-06 | ANXA2R | -4.61782 | 0.000124 |
| SLC30A10 | 8.683773211 | 0.000383 | AC118549.1 | -2.79046 | 0.000301 |
| ZNF502 | 7.58840943 | 0.000362 | GALNT4 | -2.52386 | 0.000304 |
| SELENOS | 7.171726125 | 0.000126 |  |  |  |
| OR2I1P | 7.122998739 | 5.46E-06 |  |  |  |
| AGFG2 | 6.996838543 | 6.55E-06 |  |  |  |
| TCF7L1 | 6.663929697 | 2.27E-05 |  |  |  |
| PCDH7 | 6.658245726 | 5.96E-05 |  |  |  |
| OR5AS1 | 6.204631379 | 4.57E-06 |  |  |  |

**Table S2. The top 10 up-regulation and down-regulation differentially expressed microRNAs (DEmiRNAs) between CLL patients and normal B cells**

| miRNAs(up-regulation) | foldchange | *p*-value | miRNAs(down-regulation) | foldchange | *p*-value |
| --- | --- | --- | --- | --- | --- |
| hsa-miR-4485-3p | 11.01 | 2.27E-24 | hsa-miR-409-3p | -8.3691 | 2.52E-21 |
| hsa-miR-1295a | 10.309 | 7.53E-15 | hsa-miR-485-3p | -7.4885 | 1.04E-11 |
| hsa-miR-12136 | 9.8183 | 5.76E-31 | hsa-miR-369-5p | -7.2572 | 5.22E-08 |
| novel_745 | 8.4312 | 2.47E-10 | hsa-miR-539-5p | -7.1681 | 6.95E-10 |
| hsa-miR-1973 | 8.3636 | 6.28E-11 | hsa-miR-411-3p | -6.9916 | 3.07E-08 |
| hsa-miR-1295b-5p | 8.2229 | 3.83E-09 | hsa-miR-433-3p | -6.9596 | 4.81E-09 |
| hsa-miR-4524a-3p | 7.9007 | 0.0026925 | hsa-miR-495-3p | -6.7283 | 1.09E-19 |
| hsa-miR-4524b-5p | 7.9007 | 0.0026925 | hsa-miR-412-5p | -6.7021 | 4.70E-07 |
| hsa-miR-1248 | 7.7834 | 2.98E-19 | hsa-miR-11400 | -6.695 | 6.83E-12 |
| novel_815 | 7.1753 | 8.46E-08 | hsa-miR-370-3p | -6.48 | 2.47E-14 |

**Table S3. The top 10 up-regulation and down-regulation differentially expressed long noncoding RNAs (DElncRNAs) between CLL patients and normal B cells**

| lncRNAs(up-regulation) | foldchange | *p*-value | lncRNAs(down-regulation) | foldchange | *p*-value |
| --- | --- | --- | --- | --- | --- |
| LNC_006655 | 14.22965 | 0.003398 | LNC_016367 | -11.1583 | 0.037493 |
| LNC_000120 | 13.25441 | 5.63E-06 | LNC_005840 | -11.1046 | 0.042789 |
| LNC_019940 | 13.16691 | 0.005403 | LNC_007866 | -11.0074 | 0.04066 |
| LNC_000119 | 12.96742 | 0.000161 | LNC_005152 | -10.8589 | 0.01768 |
| LNC_000255 | 12.68511 | 0.001777 | LNC_006063 | -10.8195 | 0.026695 |
| LNC_006654 | 12.48499 | 0.007811 | LNC_006889 | -10.4623 | 0.026133 |
| LNC_002026 | 12.47453 | 0.007856 | LNC_005829 | -10.309 | 0.024353 |
| LNC_019955 | 12.24366 | 0.000245 | ENST00000444958.1 | -10.3061 | 0.008191 |
| LNC_014176 | 12.1956 | 0.000525 | LNC_005877 | -10.159 | 0.028757 |
| LNC_007121 | 12.08685 | 0.00993 | LNC_005754 | -10.1583 | 0.027807 |

**Table S4.** **The top 10 up-regulation and down-regulation differentially expressed circular RNAs (DEcircRNAs) between CLL patients and normal B cells**

| circRNAs(up-regulation) | foldchange | *p*-value | circRNAs(down-regulation) | foldchange | *p*-value |
| --- | --- | --- | --- | --- | --- |
| novel_circ_0014882 | 11.363 | 0.00021 | novel_circ_0012210 | -20.745 | 2.82E-11 |
| novel_circ_0008567 | 10.992 | 0.000338 | novel_circ_0002757 | -5.9427 | 0.045319 |
| novel_circ_0011269 | 10.992 | 0.000338 |  |  |  |
| novel_circ_0002750 | 10.796 | 0.000433 |  |  |  |
| novel_circ_0008023 | 10.687 | 0.000496 |  |  |  |
| novel_circ_0011026 | 10.687 | 0.000496 |  |  |  |
| novel_circ_0011311 | 10.687 | 0.000496 |  |  |  |
| novel_circ_0001235 | 10.568 | 0.000574 |  |  |  |
| novel_circ_0004234 | 10.568 | 0.000574 |  |  |  |
| novel_circ_0006626 | 10.568 | 0.000574 |  |  |  |

**Table S5. The top 10 up-regulation and down-regulation DEmRNAs between CLL cells and normal B cells**

| mRNAs(up-regulation) | foldchange | *p*-value | mRNAs(down-regulation) | foldchange | *p*-value |
| --- | --- | --- | --- | --- | --- |
| NHP2 | 14.61655 | 0.005627 | RPL27A | -6.52876 | 0.001626 |
| XYLT2 | 10.9179 | 0.000324 | CCDC69 | -3.93153 | 0.000216 |
| TAF11L6 | 10.65921 | 1.37E-08 | COX7C | -3.83601 | 0.001258 |
| ZNF713 | 9.900218 | 7.40E-05 | DCK | -3.7552 | 0.002215 |
| CYP3A4 | 8.924985 | 0.001804 | OFD1 | -3.74183 | 0.006137 |
| TAF11L9 | 8.916205 | 4.66E-05 | ANKRD10 | -3.69217 | 0.00511 |
| FOXL2NB | 8.866608 | 5.19E-05 | UBXN4 | -3.69087 | 0.004963 |
| HSPA4L | 8.783463 | 0.000474 | TMED10 | -3.46517 | 0.004772 |
| C9orf85 | 8.530356 | 1.44E-05 | TRIM25 | -3.34463 | 0.001343 |
| TMIGD2 | 8.485102 | 8.98E-05 | SFSWAP | -3.02508 | 0.003984 |

**Table S6. The top 10 up-regulation and down-regulation DEmiRNAs between CLL cells and normal B cells**

| miRNAs(up-regulation) | foldchange | *p*-value | miRNAs(down-regulation) | foldchange | *p*-value |
| --- | --- | --- | --- | --- | --- |
| hsa-miR-4485-3p | 12.299 | 7.72E-09 | hsa-miR-584-5p | -11.922 | 7.71E-15 |
| hsa-miR-1246 | 10.842 | 2.51E-22 | hsa-miR-654-3p | -10.232 | 7.94E-10 |
| hsa-miR-3681-5p | 10.738 | 1.64E-22 | hsa-miR-4433a-3p | -9.9613 | 1.53E-15 |
| novel_900 | 9.898 | 0.009568 | hsa-miR-4433b-5p | -9.9542 | 1.61E-15 |
| hsa-miR-7974 | 9.8813 | 1.91E-25 | hsa-miR-11400 | -9.8803 | 3.70E-08 |
| hsa-miR-4517 | 9.8188 | 0.003172 | hsa-miR-494-3p | -9.4288 | 4.46E-09 |
| hsa-miR-1973 | 9.4633 | 0.013266 | hsa-miR-382-3p | -9.3616 | 1.18E-08 |
| hsa-miR-573 | 9.08 | 3.51E-12 | hsa-miR-4433b-3p | -9.2759 | 1.23E-09 |
| hsa-miR-155-3p | 9.0485 | 0.003896 | hsa-miR-4433a-5p | -9.2709 | 1.24E-09 |
| hsa-miR-138-5p | 7.7094 | 0.00076 | hsa-miR-323a-3p | -8.8588 | 2.99E-07 |

**Table S7. The top 10 up-regulation and down-regulation DElncRNAs between CLL cells and normal B cells**

| lncRNAs(up-regulation) | foldchange | *p*-value | lncRNAs(down-regulation) | foldchange | *p*-value |
| --- | --- | --- | --- | --- | --- |
| LNC_007008 | 14.9559 | 2.66E-05 | LNC_015986 | -13.6077 | 0.01111 |
| LNC_002026 | 14.12423 | 7.38E-05 |  |  |  |
| LNC_002020 | 14.0926 | 0.000722 |  |  |  |
| LNC_000335 | 13.60792 | 0.001061 |  |  |  |
| LNC_002059 | 13.56313 | 0.000138 |  |  |  |
| LNC_007018 | 13.52631 | 0.00113 |  |  |  |
| LNC_001236 | 13.4785 | 0.001179 |  |  |  |
| LNC_000602 | 13.37257 | 0.000372 |  |  |  |
| LNC_006656 | 13.29725 | 0.000179 |  |  |  |
| LNC_019482 | 12.98617 | 0.00209 |  |  |  |

**Table S8. The top 10 up-regulation and down-regulation DEcircRNAs between CLL cells and normal B cells**

| circRNAs(up-regulation) | foldchange | *p*-value | circRNAs(down-regulation) | foldchange | *p*-value |
| --- | --- | --- | --- | --- | --- |
| novel_circ_0006691 | 11.21 | 0.003484 | novel_circ_0012210 | -17.352 | 1.40E-05 |
| novel_circ_0002237 | 11.015 | 0.004123 |  |  |  |
| novel_circ_0007481 | 11.015 | 0.004123 |  |  |  |
| novel_circ_0010574 | 11.015 | 0.004123 |  |  |  |
| novel_circ_0010741 | 11.015 | 0.004123 |  |  |  |
| novel_circ_0014276 | 11.015 | 0.004123 |  |  |  |
| novel_circ_0010262 | 10.918 | 0.004449 |  |  |  |
| novel_circ_0001006 | 10.79 | 0.004998 |  |  |  |
| novel_circ_0002745 | 10.79 | 0.004998 |  |  |  |
| novel_circ_0003685 | 10.79 | 0.004998 |  |  |  |

**Table S9. Primer sequences of the genes tested by qRT-PCR**

| Gene | Primer |
| --- | --- |
| hsa_circ_0002078 | F: 5’-CAAGGAGGACACGTCGGAAC-3’  R: 5’-GGTAGCCCCGGACAATCAAC-3’ |
| hsa-miR-185-3p | F: 5’-GTAATCTTTAGGGGCTGGCTTT-3’  R: 5’-TATGCTTGTTCTCGTCTCTGTGTC-3’ |
| TCF7L1 | F: 5’-AAGTGGCACAACCTGTCTCG-3’  R: 5’-GACCAGGTTGGGTAGAGCTG-3’ |
| TRIM34 | F: 5’-TCAGGAGAAACTCCAGGCAG-3’  R: 5’-TCTTTGCAGCTCTCTCTGCT-3’ |
| SLC30A10 | F: 5’-CCTGAAGAGTGAGGACCCGT-3’  R: 5’-GCGGGAAGGCAGATGACAAA-3’ |
| HOXD4 | F: 5’-AGTATTTGCAGGGCGGCTAC-3’  R: 5’-AAAGGCTGCTCACCGAAGT-3’ |
| hsa_circ_0007675 | F: 5’-AGGACTCCACACAGACCCAA-3’  R: 5’-TGACTCTGGAATCTGCTTTGC-3’ |
| GAPDH | F: 5’-GCACCGTCAAGGCTGAGAAC-3’  R: 5’-TGGTGAAGACGCCAGTGGA-3’ |
| U6 | F:5’-CAGCACATATACTAAAATTGGAACG-3’  R: 5’-ACGAATTTGCGTGTCATCC-3’ |
